# Supplementary material for: Punctuated decline of human cooperation
Source: Nature. 2026 Apr 22;653(8116):1110–8. doi: 10.1038/s41586-026-10380-3 (PMC13215883; doi:10.1038/s41586-026-10380-3)
Supplement: Supplementary file 1 — Supplementary Methods, Tables 1–10 and references, as well as the protocol for semi-structured client interviews. [file 41586_2026_10380_MOESM1_ESM.pdf]

nature portfolio
https://doi.org/10.1038/s41586-026-10380-3

Supplementary information

Punctuated decline of human cooperation

In the format provided by the
authors and unedited

Supplementary Information for:
Punctuated Decline of Human Cooperation

Nicholas Sabin[1,2,4]*, David Klinowski[3]*, Felix Reed-Tsochas[2,4]*

[1]Universidad de Santiago de Chile, Facultad de Administración y Economía; Santiago, Chile.
[2]University of Oxford, Saïd Business School; Oxford, UK.
[3]William & Mary, Department of Economics; Williamsburg, USA.
[4]University of Oxford, CABDyN Complexity Centre; Oxford, UK.

*Correspondence to:
nicholas.sabin@usach.cl; dklinowski@wm.edu; felix.reed-tsochas@sbs.ox.ac.uk

Supplementary Methods **2**

Theoretical background 2
Codified themes and direct quotes 2
Theme 1: Cooperative dilemma structure and client understanding in the field 3
Theme 2: Extent and evidence of free-riding 4
Theme 3: Self-reported motivations for cooperation 7
Theme 4: Changes in behavior over time 10
Heterogeneity of cooperative dynamics 16

Supplementary Tables **18**

Supplementary Table 1. Group continuation status and sources of attrition across cycles 18
Supplementary Table 2. Sequential estimates of the restart effect on cooperation rates, controls included 19
Supplementary Table 3. Change in the size of the restart effect across cycles for fixed samples of groups, no controls 20
Supplementary Table 4. Change in the size of the restart effect across cycles for fixed samples of groups, with controls 21
Supplementary Table 5. Cooperation rates at the start of a cycle in group lending 22
Supplementary Table 6. Interview sample client group-level descriptive statistics 23
Supplementary Table 7. Interview sample staff descriptive statistics 24
Supplementary Table 8. Last-round effects on cooperation rates within loan cycles 25
Supplementary Table 9. Covariates of cooperation rates over time in group lending 26
Supplementary Table 10. Cycle-to-cycle transition frequency 28

Supplementary File **29**

Supplementary File 1. Protocol for semi-structured client interviews 29

Supplementary References **32**

Supplementary Methods

Theoretical background

Cooperative dilemmas are traditionally modeled as, or even equated with, a prisoner's dilemma or the more general linear public goods game – a game in which individuals decide how much of their private resources to contribute to a public good whose production is linear in the level of aggregate contributions. Free riding is a dominant strategy in this game, and individual rationality leads to a unique, socially inefficient equilibrium in which no one contributes[1,2]. However, scientists have argued that many social dilemmas are instead better modeled as an assurance game, volunteer's dilemma, or a threshold public goods game more generally, a game in which production is not linear but instead the public good is provided if and only if a certain level of aggregate contributions is reached[3–9]. Biologists, economists, sociologists, and political scientists have modeled various situations as threshold games, including environmental conservation[10], voting[11], peer punishment[12,13], group hunting, territory defense, alarm calls against predators, and enzyme production among cells[14,15]. Threshold public goods games are now widely studied theoretically and experimentally[15–25].

In a threshold public goods game, free riding is no longer a dominant strategy, since players have an individual incentive to contribute to the group if they believe their contribution will be pivotal for providing the good. Thus, in addition to zero contribution, a strategy profile in which the group contributes just enough to provide the public good is also an equilibrium, and a Pareto superior one. This does not mean, however, that a threshold public goods game reduces simply to a problem of selecting between the no-contribution equilibrium and the provision equilibrium – a coordination problem – and that it involves no element of conflict between opportunistic behavior and the collective interest. If the public good can be provided with less than full contributions from all players, then multiple provision equilibria exist, and a player prefers some provision equilibria more than others. In particular, she prefers that the public good be provided with the minimal possible expenditure of her private resources. Such a threshold game thus involves *distributional conflict*[7,26,27]. The attempt by players to achieve provision while contributing less than their fair share has been termed "cheap riding"[19]. Indeed, laboratory threshold public goods experiments find that players cheap ride, behavior which, if widespread enough within the group, will undermine provision[19,28]. It has also been observed in laboratory threshold games that players are willing to withdraw their contributions to punish cheap riding, even at the cost of failing to achieve provision[29].

Codified themes and direct quotes

The qualitative data in this study consist of 73 in-depth semi-structured interviews: 64 interviews with group lending clients and 9 interviews with members of the lending institution staff. See the Methods section of the main manuscript for ethical approval, empirical background, qualitative data overview, sample selection, data collection, data coding, and thematic analysis. Here we present the most commonly observed patterns from the interview data, organized according to the higher-order themes: (1) Cooperative dilemma structure and client understanding in the field; (2) Extent and evidence of free-riding; (3) Self-reported motivations for cooperation; and (4) Changes in behavior over time. The direct quotes are representative of the empirical patterns. We use pseudonyms in the quotes for client confidentiality.

Theme 1: Cooperative dilemma structure and client understanding in the field

Collective outcomes. The interviews allowed us to verify if the dilemma structure and incentives as practiced in the field are consistent with the theoretical framing used in this study. It is vital to the social dilemma that group members are not held individually accountable by the lending institution. The institution's policy manual and interviews with staff clearly stated that they do not differentiate how much each member has contributed and that "the program will hold each individual responsible for the entire group's loan" (Lending Institution Policy Manual).

Client interviews confirmed that this was well-understood and implemented by the lending institution:

> *"Actually before we take this loan, they teach us how to pay this money. They teach us not to only depend on one person. We need to share the responsibility of the payment" (Client Interview G6.C9).*
>
> *"Because we are told in the office that anybody in a group who fails to pay this money, we the members in the group, we are going to be responsible of paying it" (Client Interview G13.C23).*
>
> *"So they decided to leave the group and we were responsible for paying this money" (Client Interview G32.C59).*

Awareness of group member behavior. The extent that group members are aware of each other's behavior affects game theoretic strategy and incentives. For example, if the clients are often unaware of the group's ability to reach the collective payment each month, coordination would become a strategic concern. Additionally, if members are not aware of which group members had contributed in the prior round, reputation would serve as less of an incentive.

In general, clients reported high levels of interaction and awareness of group members' behaviors.

> *"In fact, I usually pay a visit to them very fast, every day. And when it is time for me to pay this money, we will get ourself very close together and discuss about this money" (Client Interview G6.C10).*
>
> *"We see [each other] every blessed day. Except when a member of the group is sick and decided to go to her home" (Client Interview G22.C43).*
>
> *"We will know because we see, and we meet every week…. We make jokes, talk about the family, and we talk about the micro[finance], and we talk about the business…. If we meet around 5:00, we spend until 7:00 o'clock before we depart" (Client Interview G2.C4).*
>
> *"Every day we see [each other], because we stay in the same community. And our houses are within the same community…. When it is time for this money, we come and sit down and discuss about the money, and if I have a business, I will call her*

so that we share ideas of business. She too has a business, and I too call and share the same ideas, we share business ideas together" (Client Interview G9.C16).

Less commonly, some groups reported infrequent contact, such as once or twice a month (Client Interviews G4.C7, G7.C11).

Equal division and distributional conflict. In a group loan, all members receive the same loan amount and are aware that they are equally responsible at the outset. Interviews confirmed that members understood the loan amounts and equal division. In practice, clients keep track of who has paid and frequently differentiate between paying their own share versus paying for another member's share.

"[Fatmata] paid all the money but [Haja] has left about 80,000" (Client Interview G13.C23).

"So I myself will not come and say, 'you pay for me,' then I hold the money without paying it. So I have to settle my own problem, they too have to settle their own problem" (Client Interview G14.C27).

The distributional conflict arises as members often attempt to underpay their share but hope to remain in the group and retain access to future loans.

"We paid for her because that's what we have agreed on. If any one of us have a problem, we need to contribute and pay for that person" (Client Interview G9.C17).

In other groups, the distributional conflict results in a breakdown of cooperation.

[Interviewer: Are the other members contributing for the defaulting member?] "No. The one said she is only trying to pay for herself" (Client Interview G11.C20).

Theme 2: Extent and evidence of free-riding

Types of free-riding and their frequency. Free-riding occurs when those who benefit from a collective good do not contribute or under-contribute[30]. In the context of group lending, the collective good is principally access to future loans. To what extent do clients not contribute their share of the group loan and maintain access to future loan disbursements?

All client interviews directly addressed the extent, cause, and impact of members not fully contributing. In this section, we present the summary of coding these behaviors. The frequency of free-riding behaviors are informative because they are drawn from a representative sample. Extended Data Table 5 provides coded free-riding behavior for each group. We first quantify the extent of borrowers not contributing their share. We then quantify the other members' responses in terms of their willingness to compensate for the defecting member and, finally, their choice to include or exclude the defaulter from future loan access.

We find no evidence of full free-riding (0.0% of interviewed groups), operationalized as groups with at least one member that receives their share of a loan and makes zero contributions. We find extensive evidence of partial free-riding (91.4% of interviewed groups), operationalized as groups with at least one member who receives their share of the loan and does not make full contributions,

all months, and on-time. Infrequently, we find groups with no evidence of free-riding (8.6% of interviewed groups), operationalized as groups where all members make full contributions, all months, and on-time.

Partial free-riding in group lending is consistent with the theoretical concept of cheap riding in the threshold public goods literature, such that agents hope to achieve collective provision while contributing less than their fair share[19]. Within the category of partial free-riding, there is a spectrum of behaviors in our setting.

Heterogeneity in partial free-riding behavior. Interviews show substantial variation across four variables: (1) amount unpaid, (2) compensating for the defecting member(s), (3) reciprocity, and (4) treatment of the defecting members.

(1) Variation in amount unpaid. In many cases, a member may skip one or two monthly payments.

> *"We have assisted [Isha] once" (Client Interview G2.C4).*
>
> *"They are not completing the money. It's about two months they are not paying" (Client Interview G13.C23).*
>
> *[Interviewer: How often did you have to compensate for another member?] "It was only this past month…. We are not embarrassed" (Client Interview G21.C42).*

Less commonly, members defect on several payments.

> *"We had only paid for three months and she takes off"[five of eight payments remaining] (Client Interview G3.C5).*
>
> *"The only thing I can tell you here, she just decided to eat the money…. She only paid for five months. [long pause] She goes fast, she run away, and then later she come again, she come back, and pay three months…. So it remains two months for the first cycle" (Client Interview G24.C48).*

Infrequently, groups had members that defected on most of their payments, for example, 9 out of 10 payments (Client Interview G11.C20).

(2) Variation in compensating for a defecting member. All groups agree at the outset that they will be collectively responsible for each other. However, as the loan progresses groups differentiate in their willingness to compensate for a defecting member. Here, we see two different groups with a comparable situation: a death in the group member's family. The cooperative response was opposite in the two groups.

> *"We pay late because one of our members has a funeral. She lost her family member…. Well, since we do not see her, we come together as a group and pay for her. That's why we are late" (Client Interview G4.C6).*
>
> *[Interviewer: Did anyone in the group help you to pay?] "No! [client laughing] Even if your father is dead and the corpse lying down with us…. they will not pay for you. Ah hah" (Client Interview G20.C40).*

(3) Variation in reciprocity. Clients report very different expectations for reciprocal behavior. Some compensate for others with no expectation of reciprocity, while others compensate with clear expectations to be paid back or assisted later if they encounter a difficulty in repaying.

> *"Others pay half of the money back, but others no way" (Client Interview G25.C50).*

> *"We decided to help. I decided to pay for him so that we will not disturb our program." [Interviewer: Did Abdul pay you back?] "Yes…. Even though he pay bit by bit, I told him I am paying because I don't want to disorganize the group. Because he do not used to disturb us" (Client Interview G7.C11).*

(4) Variation in treatment of a defecting member. When a member defects, they have already received the financial disbursement in full for the current loan cycle. It is the choice of the group whether a free-riding member will be allowed to participate in the next loan cycle and continue to receive disbursements. The group's choice is often based on the perceived motives for defection, frequency, attitude, and process of communication. Some groups will even decide to allow one defector to remain in the group and another to be excluded.

> *"When we finish paying, we will kick [Memuna] out…. She is not serious in paying…. she just decided to go away and leave us paying this money. So I used to feel bad about it" (Client Interview G6.C10).*

> *"[Ramatu] failed to pay…. That is the problem with traitors. They hardly give out money…. And she came with this money today. And she pleads to me, says 'Please [Hawa], forgive me.'" [Ramatu was allowed to stay in the group] (Client Interview G5.C8).*

Frequency of compensating for and treatment of a defecting member. For groups reporting free-riding behavior, we report the frequency of group members compensating for the defector and the resulting treatment of the defector. Extended Data Table 5 shows the coded results of compensating for a defector and excluding a defector.

We found that 65.7% of groups reported compensating for at least one defector, coded as at least one member of the group having reported paying some portion of another member's share. However, 37.1% of groups reported not compensating for at least one defector. Note that these percents total more than 100% because these behaviors are not mutually exclusive: groups with multiple free-riders may compensate for one defector and not for another.

How often was a defector allowed to stay in the group and access the next loan cycle disbursement? We found that 57.1% of groups reported allowing a defector to remain in the group. However, 48.6% of groups reported excluding a defector from the next loan cycle. Again note that these percents total more than 100% because these behaviors are not mutually exclusive: groups with multiple free-riders may allow one defector to remain and exclude another.

Theme 3: Self-reported motivations for cooperation

Substantial heterogeneity in self-reported motivations. All interviews discussed the clients' motivations for cooperating. Answers range from purely financial to prosocial. As these motivations are self-reported, they should be interpreted in light of potential social desirability bias. However, we believe the patterns are nevertheless informative because they are often embedded in a larger narrative of their specific group experience. In addition, clients are not restricted to selecting only one motivation. For example, clients may report being motivated for access to future loans as well as avoiding social embarrassment.

In order to help communicate the results, we code the responses into broad motivational categories: (1) economic, (2) duty/morality, (3) reputation/embarrassment, and (4) solidarity/trust. Conceptually, these categories could be much more nuanced. For example, solidarity can be theoretically distinguished from different forms of trust. However, we did not want to overinterpret free-form answers and imply theoretical precision that is not really there. We do think that these four categories represent meaningful differences in what the clients were trying to communicate.

All answers are in response to some variant of the question "Why do you choose to repay the loan?" In this section, we first provide representative quotes and the frequency of motivations in regard to why a member reports paying their own share. However, a notable theme that emerged from the data was the difference in motivations regarding why a borrower pays their own share versus why they pay for another member's share. We accordingly structure the presentation of the interviewee quotes to account for this distinction in the text and coding Extended Data Table 4.

(1) Economic. Having already received the loan disbursement, the economic incentive for repayment is primarily access to future loans and is commonly reported by clients.

> *"I want more money. I want so that they will give me more loans. I don't have anybody who can give money to me" (Client Interview G21.C41).*
>
> *"They say if you fail to pay, you might misuse your [financial] opportunity" (Client Interview G22.C45).*
>
> *"I choose to pay this loan so that they will give me another loan" (Client Interview G4.C7).*
>
> *"If we do not pay complete, they will not give us the other loan" (Client Interview G6.C9).*
>
> *"The more you pay, the more they increase the loan. And I want to do a business" (Client Interview G13.C23).*

(2) Duty/Morality. By contrast, some clients reported motivations related to more abstract principles such as duty, obligation, or morals.

> *"Except death, or I was seriously sick, that will lead me not to be able to pay this money. But as long as I am healthy and I am still alive, I will pay this money. It's my responsibility to pay it" (Client Interview G14.C28).*

> *"It is a sin [not to pay]. Actually, how will you expect somebody to trust you the money and share this money within your group, and you decide not to pay?" (Client Interview G5.C8).*
>
> *"It is my own responsibility for me to go and meet with everybody [to pay]" (Client Interview G22.C43).*
>
> *"Well, we don't have an alternative…. We collect the money from each other and we go together as a group and then we pay" (Client Interview G19.C38).*
>
> *"If you swallow it, you need to spit it out. No matter, so you must have to pay" (Client Interview G15.C29).*

(3) Reputation/Embarrassment. Another commonly reported motivation focused on social reputation and the avoidance of shame and embarrassment.

> *"I am always afraid of embarrassment because this is happening several times with our [neighbors]" (Client Interview G5.C8).*
>
> *"Even the embarrassment, you fail to pay, it is a disgrace" (Client Interview G22.C45).*
>
> *"At the initial stage I was not happy, because I feel embarrassed. To see all my colleagues in other groups paying their money, and I am not paying. I am not feeling good about it" (Client Interview G11.C20).*
>
> *"It's a pride for me. Even if I am playing with this money, it's a big pride…. I am doing my business. I have my own dress there, I take care of my family, and of course I also have respect within my community" (Client Interview G5.C8).*
>
> *"[We went to the loan office] ….and then I tried to talk to the cashier so that he can understand, and he said let me just keep waiting…. I am always ashamed…. I never get such embarrassment like this" (Client Interview G30.C57).*

(4) Solidarity/Trust. The final motivational category relates to solidarity and trust, potentially even prosocial help and inequality aversion.

> *"She trusts me and I trust her. And she knows I will not run away with this money" (Client Interview G16.C31).*
>
> *"Actually I rely on them…. I don't think it fits to say, 'let them pay for me.' And I will always work hard so that I will pay back this money. And they too rely on me" (Client Interview G21.C42).*
>
> *"I don't want to fail my group. That's why I pay it" (Client Interview G26.C53).*
>
> *"It is not easy for them to pay for me. Even I traveled to [City Y] once upon a time. I travelled to [City Y]. When it is time for payment, I rushed to come to back [home] so that I will pay" (Client Interview G10.C18).*

"Because if I don't pay, our members of the group undergo the same constraints. So I don't want them to strain" (Client Interview G2.C4).

"In fact we all strain…. Yes, we all have some constraints. We are trying to make things work very easy for us, so I will not leave my problem with them" (Client Interview G2.C3).

Extended Data Table 4 details the coding for each of the 64 client interviews and a summary of the frequency for each of the self-reported motivations. 34.4% of interviewees reported an economic motivation for why they pay their own share. The concern for reputation and avoidance of embarrassment was reported at a similar frequency of 40.6%. The most frequently reported motivations for paying one's own share were solidarity and trust at 75.0% and duty and morality at 68.8%.

Distinction in motivations for paying own share versus other's share. The structure of a social dilemma with a collective threshold implies that self-interested participants should prefer that the collective good be provided whilst the individual contributes as little of their personal resources as possible[26,27]. This distributional conflict encourages participants to keep track of how much they have paid in comparison to other members. In the group lending context, interviewees strongly distinguish paying their own share from paying another's share and systematically reported different motivations for paying what they viewed as another member's share (Extended Data Table 4). Whereas the motivations for paying one's own share are diverse, ranging from economic to a variety of social motivations, the motivations for paying another member's share are comparatively narrow. They focus almost exclusively on solidarity and trust at 34.4% and duty and morality at 21.9%. Economic motivation was never reported as a reason to pay for another member's share, even though it may be rational given their shared financial fate. Concern for reputation and avoidance of embarrassment was only reported by 6.3% of clients as a reason to pay for another. Here the quotes show a shift towards solidarity thinking.

"Actually we have a group, a community group we are in. If any one of us gets sick or has any problem, we contribute and then we meet this person and try to assist where we can. And we normally used to counsel each other about our business" (Client Interview G9.C16).

"We love ourselves…. If any one of us has a job to do which is very difficult, we meet, we call, we mobilize ourselves and assist each other" (Client Interview G14.C26).

"If someone is not financially strong, we used to call each other and try to put moralities in place to see how best we can assist" (Client Interview G14.C28).

"We do not help you freely, we help you so you do not destroy the image of our group" (Client Interview G21.C42).

"You know business is a rise and fall. At times when you bring the business, it sells very fast. But when it happens when it goes very slow and we are about to go and pay, so we have to assist the individual" (Client G2.C4).

The interview data indicate that the majority of self-reported motivations for cooperation are related to social norms and preferences. Such motivations are known to be sensitive to context and norm salience[31–33]. The following theme specifically examines the decay in cooperative motivation and behavior over time.

Theme 4: Changes in behavior over time

The interview data provided insight to behavioral mechanisms that underly a decline in cooperative performance over time. The results are organized into five subthemes: the relative role of financial inability to pay in collective outcomes; borrower's attention to distinguishing legitimate financial inability from behavioral choices; decline in cooperative motivation and cooperative effort over time; the persistence of behavioral decline in the long-term; and attempts to reduce or reverse the decay in cooperative motivation.

The relative role of financial inability to pay in collective outcomes. The interview data confirm the expectation that group lending clients often find it financially difficult to pay their share of the loan and at times have legitimate reasons for not being able to pay, e.g. a house fire (Client Interview G22.C43), unexpected medical bills (Client Interview G9.C16), slow business (Client Interviews G5.C8, G1.C1), or theft (Client Interview G7.C11). However, the data also suggests that variation in a group member(s) inability to pay is not the principal cause of the longitudinal pattern of punctuated decline.

When group members encounter legitimate financial difficulties, other members are often willing to compensate to make the full payment on time or coordinate to quickly make a partial payment to the lending institution. This happened regularly for both minor and more severe financial challenges.

> *[Multiple members of group G22 lived together in a house that was destroyed by a fire during the last month of their loan repayment. Other group members compensated and the group was able to complete their loan payment only three days late.] "I live at Number [62] on [Market Street]. My house was destroyed by fire…. So we decided to, we used to pay the 25th, but because of this accident now we decided to pay the 28th" (Client Interview G22.C43).*
>
> *"I was sick and even my baby, he too had to undergo an operation.... That is why we are always working as a unity…. We try to come together and try to assist each other. Then we complete the money and pay" (Client Interview G9.C16).*
>
> *"I will call them in a meeting. When I call them to a meeting, I will explain to them that my business is not going fast. What are we going to do? And then one of them will say, well, since the business is not going too fast, one of them will sacrifice and say, 'let us just try to – I will pay,' so that we will not have problem to go and pay" (Client Interview G1.C1).*
>
> *[Several members of Group G5 buy items from Guinea and resell them in Sierra Leone. An unfavorable change in the exchange rate was making their business more difficult. But another member of the group applied pressure to maintain complete*

and timely repayment.] "We all hope in Guinea to get some good materials there that we can do our business with. But since there is a problem in Guinea, we are hesitating. Things are going very expensive there…. We used to do the exchange of this dollar about 400,000. Now it is 450,000…. They are crying about the dollar in Guinea, and that is the only problem…. I ask them let all of us go and take the money…. But if they become very reluctant to pay, I will become very hard with them…. And we always pay on time" (Client Interview G5.C8).

Borrower's attention to distinguishing legitimate financial inability from behavioral choices. Client descriptions demonstrate that groups often sustain cooperation in the face of legitimate financial challenges, but are aware of the difference when members' behaviors become "stubborn" (Client Interview G18.C36), "lethargic" (Client Interview G17.C34), and "relaxed" (Client Interview G2.C3). Group lending clients in Sierra Leone are in a unique position to distinguish legitimate financial difficulties from behavioral choices because the groups are typically highly socially embedded. As noted previously, there is variability, but group members often meet regularly and are well-aware of each other's business and social behavior in the community. The result is that group members are well-positioned to assess the validity of a defector's explanation.

"We know because we are doing the same - we are doing our business within the same area. So if someone is around and we normally look at the business, if the business is not going too fast we are able to detect it because we stay in the same market area. So we are able to know those whose business are going very fast and those whose business they are not moving very fast" (Client Interview G14.C28).

[Client explaining that she knows that another member has not been using the money as intended for business purposes.] "When they give the money to her, she misused the money…. She used it as a family money, just to feed the family" (Client Interview G4.C6).

[Three group members work nearby in the same market all engaged in petty trading. One member stops paying and says it is because of lack of money. But the other members do not believe the excuse.] "When it's time to go and pay this money we ask [Ester], she says she don't have money…. I don't believe her…. I am not the father or not mother of [Ester]…. But she really disturb us in paying." (Client Interview G12.C22). [The other group member who works in the same market independently confirmed the assessment.] "[Ester] is tired of paying…. We kicked her out because she is very stubborn to pay…. But I used to know her to be someone who is very serious. It is just unfortunate she turned the other way, failing to pay the money…. We used to see her in the market, but now she is not in the market anymore…. since she was embarrassed for this money"(Client Interview G12.C21).

Staff experience with joint-liability groups also indicated that understanding non-contribution requires looking beyond clients' stated explanations and considering additional forms of evidence. They observed that clients often provide accounts that minimize their own agency, portraying their decisions not to contribute as less voluntary than they may actually be.

"So, normally, they will say, 'oh, my business has failed' or 'somebody stole my money,' things like that. Or 'I went to the market and I lost the money on the way,'

you know? So many excuses… Mostly those who say 'no,' [it] is just because they don't want to pay. They just don't want to" (Staff Interview S3).

"They complain that the sales are not going fast the most, we are not getting sales…. That's the way they normally complain. But when you look at all their business, actually, it's very good. Very good looking" (Staff Interview S4).

Decline in cooperative motivation and effort over time. The interviews indicated that such changes in behavior often cause groups to struggle. While uncooperative behavior can arise at any time, many client descriptions indicated a decay in cooperative motivation and effort as the loans progressed. The decline affected both the motivation to pay one's own contribution and the willingness to compensate for another member.

"Even this second cycle, we didn't at the initial stage, we didn't have any problem…. The last two months, people have become very lethargic to pay this money." (Client Interview G17.C34).

"The others have decided to relax, not paying the money, and we cannot find [them]" (Client Interview G2.C3).

[Near the end of the first loan cycle, a member of Group G18 became reluctant to pay.] "[The problem] is only [Kadiatu]. She only become very stubborn to pay once…. I felt very bad. And then we wait for her…. Of course we have met as a group and we discussed about it." [The other members applied informal pressure and the defector decided to contribute within the week] (Client Interview G18.C36).

[Group G32 was nearing the end of its second loan cycle with two months remaining when two of the group members stopped contributing.] "They are not, they are not hard-working…. I think they are not responsible. They show irresponsibility in their mind." (Client Interview G32.C59).

"At times when you go there, you get fed up with asking the person to pay, to pay, to pay" (Client Interview G18.C37).

"Initially, they have the kind of zeal, motivation to repay… But then as the time progresses…. it becomes very much boring…. So they begin to find it difficult to repay" (Staff Interview S7).

The accounts reveal several tendencies associated with decreased motivation to contribute to the group loan. Some descriptions show explicitly that the defecting member had chosen to allocate their potential repayment share towards self-interested uses at the cost of burdening the other group members. In other examples, defectors are not able to provide a valid explanation – from the perspective of the other group members – for non-contribution. The accounts consistently highlight that such changes were typically not precipitated by unexpected exogenous shocks, but rather seem to result from a shift towards immediate self-interest. Across interviews, the choice to defect was often associated with reduced concern for the other members and reduced effort in maintaining positive social relationships.

[A member paid the first loan cycle completely, but became resistant to contribute during the second loan cycle.] "At the initial stage she appeared to be someone who is serious in terms of payment. But why is it that we continue to do the same thing and she just changes her behavior? She becomes reluctant to pay…. I can't tell [why] because I always went there [to her house] to ask for the money and she don't give me any good reason." (Client Interview G35.C64).

[A member of Group G11 had defected on her last three payments.] "Normally she gives confidence to us…. So that's what I'm expecting from her." [However, the defecting member was avoiding communicating with the group. The primary sentiment of the interviewee was an emotional response to the change in social behavior.] "[Sallay] disappointed me. And it's like saying it's a great big disappointment to me" (Client Interview G11.C20).

"When we start this, we receive this money, we share the money to ourselves. Then later…. [for] two months [Adama] was not responsible of paying it…. What she did here is, she kept all this money and made use of it in a different situation…. We met in [Adama's] house to discuss about it…. She tells us the truth" (Client Interview G19.C38).

"They rather prefer to buy dress, cloth dress, so that just to decorate their body. They live in world of fashion" (Client Interview G25.C50).

[Regarding the change in social relationship with a defecting group member….] "With all the struggle that she left with us, I will not even talk to her" (Client Interview G23.C46).

"That's what makes us not to pay…. we have childrens (mm hmm) for our school affairs. And when we look at the situation…. buying books and others, we rather prefer to use that money" (Client Interview G9.C17).

"So they start using the repayment funds…. for themselves. They use it to make an advantage, you know…. 'I will make more money….' It's natural for people. Once you start making money, you want to make more money" (Staff Interview S2).

[Several members of Group G30 were previously close friends. But during their first loan cycle there was a notable change in attitude such that the defectors did not even inform the other members that they did not intend to contribute.] "I took Zainab to be [like] a sister. She is a friend to me, but a sisterly friend…. I took them to be people who are somewhat serious, but they just start to pay and then they run away…. [They] decided to abscond…. I can't find them" (Client Interview G30.C57).

The persistence of behavioral decline in the long-term. The interview sample includes interviewees from across all loan cycles. We found that changes in cooperative motivation and reduced effort were also likely to emerge in later loan cycles even after years of successful cooperation.

[A group had been cooperating over multiple loan cycles. In the fourth loan cycle, two members decided to stop contributing and intentionally deceive the other group members.] "They only paid for five months [of the fourth loan cycle]…. Actually, they did not inform me. All that I knew, I try to call them through the phone. When I call them they used to tell me lies, saying 'Let me just go and sit down at [the loan office].' They are on the way coming…. they will come and give me something very kind. So that I will replace what they have to pay. But they keep on lying to me, then I feel disappointed about it" (Client Interview G27.C54).

[In the fifth loan cycle some members became reluctant to pay and were "kicked out" of the group.] "They are not all of them regular in payments…. Some of them, they bring their money, they pay their money completely. Why should [she] not pay?"…."So this is the embarrassment and this horribleness of my problems…. We finish paying it…. After paying the money, then we decided to kick [them]out" (Client Interview G25.C50).

[After cooperating for four loan cycles, Group G33 started to decline in the fifth cycle. When asked why in the interview, a member had no specific justification.] "If we have to pay for today, we went to the office and pay. That's the whole story… We make sure we pay everything." [However, they had just paid 25 days late. After reflecting, she then added that the group needed more motivation.] "They [the loan office] need to encourage us more to pay." (Client Interview G33.C60).

Related research in this context has shown that motivation also plays a role in the willingness to enforce social sanctions within joint-liability groups[34]. Rather than the willingness to contribute financially to the first-order dilemma, willingness to contribute to compliance enforcement among non-contributors has been conceptualized as a "second-order cooperative dilemma"[35].

Attempts to reduce or reverse the decay in cooperative motivation. Many clients informally attempt to slow the decay in cooperation throughout the loan cycle. More formally, the lending institution "resensitizes" clients (see description below) at the beginning of each new loan cycle in an attempt to reset cooperative behavior. To combat cooperative decay, both clients and staff emphasize two aspects of cooperative motivation: the financial consequences of successfully repaying, which may revive long-term self-interest, and the social obligations to fellow group members, which may draw on more emotional and prosocial sentiments. The examples highlight the role of repetition in maintaining motivation. Stating the consequences and obligations once is not enough. The efforts to slow down the decay in cooperation produce varying degrees of success at the group- and aggregate-levels.

[A staff member recounting how successful groups maintain cooperative motivation.] "'If we pay on time without any lateness or whatever, our loan amount is going to be increased for the subsequent loan…. So please, gentlemen or ladies, let's try and make sure that we pay within time….' So that's what they'll do. They'll do it initially immediately when they receive the loan, but then they will have go back to their various businesses…. [In later months] a member will pass around for the good groups and tell them, 'Please, let's make sure that we do this thing so that we'll access higher loan sizes in subsequent times'" (Staff Interview S7).

"I always keep informing her [group member] about this money that we took, and I continue to say we must work very hard to pay this money, otherwise they will not give us another loan" (Client Interview G35.C64).

"I encourage them, I pay a visit to their business, and I continue to talk to them" (Client Interview G1.C1).

"We have sympathy to each other. We normally encourage each other, I encourage my, even my members, I am calling them to pay this money" (Client Interview G17.C35).

"They often start out paying very well. First month well. Second month well. Third well. Fourth, fifth, they start a small problem…. a little late, a little late. And then they always get later towards the end of the loan…. I think that's just fatigue. Fatigue of paying back"(Staff Interview S8).

"I encouraged them… advise them so that they will pay back the money…. When I used to collect this money from my colleagues, I used to talk to them, smile to them, and laugh with them so that I receive the money without any problem" (Client Interview G22.C43).

"Praise God, she has paid completely…. In fact, she used to delay in paying…. We went there [her house]…. About three or four times. About four times now we paid a visit…. Which is almost for any month, we went there…. [Eventually] she paid…. except she had to work very hard to pay it back" (Client Interview G12.C21).

"They begin to drag their feet…. when you think if they're in their sixth cycle, you know, then these would be very good clients…. [but] they begin to just be very much lazy…. So we [resensitize] them…. 'If things continue like this, we'll drop you off….' and that will, like, give them a rethink that, 'Oh, I should stop this….' So they will catch up again" (Staff Interview 7).

[Formal excerpt from the resensitization process as described in the lending institution's policy manual.] "Review solidarity group lending principles with all group members for second cycle and higher loan applications. Ensure all members clearly understand group solidarity lending…. [The loan officer] facilitates a discussion about solidarity group lending, including the rights and responsibilities of group members…. Solidarity group lending - loans are guaranteed by the group as a whole; all the group members should know and trust each other. Note that obtaining larger loans requires excellent repayment. The program will hold each individual responsible for the entire group's loan. On each repayment date, each group member will need to immediately pay for any members that are late with their repayments" (Lending Institution Policy Manual).

"Actually the difference that I saw here is normally there are some other members of the group, before the ending of the month, they will just go instantly and…. they would give the money…. While others, you keep reminding them, you keep

reminding them, they will not pay. So this is how this thing normally happens" (Client Interview G25.C50).

Heterogeneity of cooperative dynamics

Extended Data Table 6 presents heterogeneity within-groups at the level of individual contributions and summarizes the frequency of different cooperative patterns based on the interview data. Recall that we conducted interviews with a representative sample of 35 joint-liability groups (consisting of 158 group members). The cooperative patterns refer to the group's current or most recent loan cycle at the time of the interview to minimize recall bias. The client descriptions were cross-validated with secondary interviews and group-level repayment records from the lending institution. See Figure 4 for the sampling methodology and Supplementary File 1 for the full interview protocol.

To identify the predominant cooperative pattern in a group, we followed a two-step strategy. In Step 1, we classified each member of each group into an individual behavior type based on the defection behavior reported in the interviews. Then, in Step 2, we categorized groups into various cooperative patterns based on an aggregation of the individual behaviors coded in Step 1.

Step 1: Coding of Individual Behavior. To capture the heterogeneity in the dynamics of cooperative behavior within a group, we classified each member of each group into an individual behavior type. Across all cases, we classified a member as exhibiting "defection" if the member was reported as unable or unwilling to contribute their share of the loan in any given round (month). More specifically, we classified different patterns of defection as follows. We classified a member as exhibiting "walk-away defection" if the individual was reported as defecting for more than one round and not contributing again during the loan cycle. We classified a member as exhibiting "intermittent defection" if the individual was reported as defecting for one or more rounds but later contributing in a subsequent round of the loan cycle. If a member was reported as defecting only in the last round of the loan cycle but staying in the group to contribute in the next loan cycle, we coded this individual as exhibiting intermittent defection. Finally, we classified a member as exhibiting "no defection" if there was no report that the individual was unable or unwilling to contribute their share of the loan in any round (month).

Step 2: Categorization of Group Cooperative Patterns. We defined categories to capture substantive differences in within-group repayment dynamics described in the interviews. For each group, we aggregated the group members' individual behavior types coded in Step 1 into a group cooperative pattern based on the predominant pattern observed. We use capital letters to denote group patterns in order to distinguish them from individual behavior types, which are denoted with all lowercase letters. We classified a group as exhibiting "No Defection" if all its members exhibited no defection. We classified a group as exhibiting "Walk-Away Defection" if one or more of its members exhibited walk-away defection and one or more members continued to contribute. In the case of multiple walk-away defections in the group, they all must have been reported to have started in the same round for the group to be classified as Walk-Away Defection (in contrast to a Cascade). We classified a group as exhibiting a "Cascade" if multiple members exhibited walk-away defection that were reported to have increased over rounds (months), and one or more members continued to contribute (in contrast to Full Collapse). We classified a group as exhibiting "Intermittent Defection" if one or more of its members exhibited intermittent defection and no

members exhibited walk-away defection. Finally, we classified a group as exhibiting "Full Collapse" if all its members end up in defection. For two groups (G1 and G4), some of its members exhibited intermittent defection and others exhibited walk-away defection; we classified both groups as Walk-Away Defection to reflect the more severe defection behavior.

Results: Frequency of Group Cooperative Patterns. Consistent with the quantitative analysis of the full dataset, we found reports of individual defections increasing over rounds within loan cycles. Underlying this trend, there is heterogeneity in the severity of the defections (Extended Data Table 6). The most prevalent pattern was Intermittent Defection reported in 62.9% of groups. Defectors in these groups maintain some degree of cooperation throughout the loan cycle. Group members' responses to intermittent defectors vary substantially. Such behavior may allow the defector to retain access to the collective good, result in exclusion from the subsequent loan cycle, or cause the whole group to lose access to future credit.

Groups exhibiting "Walk-away Defection" behavior represent 17.1% of the qualitative sample. In groups that exhibited Walk-Away Defection, members who did not defect often indicated that they continued to contribute primarily out of regard for the remaining members or a sense of obligation (e.g. G11.C20; G23.C46; G28.C55). We found that "Cascades" of walk-away defections occurred in 8.6% of groups. If groups proceeded to the next loan cycle, walk-away defectors were often excluded from the next cycle at the discretion of the group members. The interviews showed that even when groups experienced walk-away defections or cascades, most groups had remaining members that continued to contribute to the group loan, with only 2.9% of groups reaching full collapse in which all members defected.

A few additional important observations emerge from the analysis of within-group dynamics. Interestingly, the vast majority of groups exhibit some pattern of defection, with only 8.6% of groups exhibiting No Defection. These results provide important nuance that would not be apparent from the quantitative results alone, for which we observed that most groups repay in full in any given loan cycle (Figure 1, Extended Data Figure 1). If we generalize the distribution of group cooperative patterns observed in the interview sample to the full quantitative sample, the results in Extended Data Table 6 suggest that even when a group repays in full, a group is likely to experience some tension or conflict among members. Thus, full repayment by a group does not imply, and likely often does not involve, full cooperation by all its group members. This emphasizes the value of our alternative cooperative effort rate measure and of our qualitative analysis. Finally, we note that the percentage of groups that exhibit Full Collapse in the qualitative sample is in line with the percentage of groups in the quantitative dataset that end with zero percent financial contribution rate in any given loan cycle (Extended Data Figure 1).

Supplementary Tables

Supplementary Table 1. Group continuation status and sources of attrition across cycles

	Cycle 1	Cycle 2	Cycle 3	Cycle 4
Group continues	64.57	72.22	62.35	42.64
Group does not continue				
Full loan amount repaid				
Average repayment on time	7.99	3.51	8.10	13.42
Average repayment not on time	1.45	0.58	4.05	5.19
Full loan amount not repaid	5.35	13.35	23.35	33.77
Continuation status truncated	20.64	10.33	2.16	4.98
Number of groups	1589	1026	741	462

Notes: Percentage of groups in a cycle that renew their loan for a subsequent cycle or drop from the sample for the reason specified in the table. Groups were categorized as "average repayment on time" if their days overdue within the 30-day window did not exceed a week on average for the loan cycle. "Continuation status truncated" refers to groups that completed their loan cycle near the end of the data collection window and their continuation status is unknown.

Supplementary Table 2. Sequential estimates of the restart effect on cooperation rates, controls included

A. Financial contribution rate	1st cycle & 1st restart	2nd cycle & 2nd restart	3rd cycle & 3rd restart	4th cycle & 4th restart	Overall
Mean round-to-round change	-0.036	-1.027	-2.043	-0.785	-0.760
	(0.258)	(0.438)	(0.383)	(0.290)	(0.100)
	[p=0.8882]	[p=0.0189]	[p<0.0001]	[p=0.0069]	[p<0.0001]
Mean change at restart	-0.327	5.467	15.903	6.818	4.380
	(1.373)	(2.579)	(2.930)	(2.541)	(0.620)
	[p=0.8118]	[p=0.0340]	[p<0.0001]	[p=0.0073]	[p<0.0001]
Diff in means (restart effect)	-0.291	6.494	17.945	7.603	5.140
	(1.631)	(3.016)	(3.313)	(2.831)	(0.719)
	[p=0.8585]	[p=0.0313]	[p<0.0001]	[p=0.0072]	[p<0.0001]
Controls	Y	Y	Y	Y	Y
N groups	1,026	741	462	197	1,026
N obs	6,494	5,108	3,994	1,922	17,518
R^2	0.0151	0.0362	0.0747	0.0292	0.0197
**B. Cooperative effort rate**	**1st cycle & 1st restart**	**2nd cycle & 2nd restart**	**3rd cycle & 3rd restart**	**4th cycle & 4th restart**	**Overall**
Mean round-to-round change	-0.866	-2.473	-1.564	-0.919	-1.187
	(0.303)	(0.511)	(0.325)	(0.315)	(0.104)
	[p=0.0043]	[p<0.0001]	[p<0.0001]	[p=0.0035]	[p<0.0001]
Mean change at restart	0.150	7.959	11.078	7.050	3.227
	(1.616)	(3.009)	(2.482)	(2.757)	(0.650)
	[p=0.9259]	[p=0.0082]	[p<0.0001]	[p=0.0105]	[p<0.0001]
Diff in means (restart effect)	1.017	10.433	12.642	7.969	4.414
	(1.919)	(3.520)	(2.807)	(3.072)	(0.754)
	[p=0.5963]	[p=0.0030]	[p<0.0001]	[p=0.0095]	[p<0.0001]
Controls	Y	Y	Y	Y	Y
N groups	1,026	741	462	197	1,026
N obs	6,494	5,108	3,994	1,922	17,518
R^2	0.0157	0.0458	0.0620	0.0560	0.0211

Notes: Predicted restart effect estimated from OLS regressions of the group's round-to-round change in the financial contribution rate (Panel A) or cooperative effort rate (Panel B). The sample includes observations only from rounds that are part of a restart. For example, "1st cycle & 1st restart" compares the average round-to-round change in cooperation rates for all rounds in cycle 1 ("Mean round-to-round change") to the change in cooperation rates between the last round of cycle 1 and the first round of cycle 2, when the sample includes only groups that continue from cycle 1 to cycle 2. Regressors are an indicator for the restart round and groups fixed effects. All regressions control for loan size, loan duration, calendar year fixed effects, and rainy season fixed effects. Standard errors clustered at the group level in parentheses, and p-values from two-sided t-tests of the coefficient estimates in brackets.

Supplementary Table 3. Change in the size of the restart effect across cycles for fixed samples of groups, no controls

	A. Financial contribution rate			B. Cooperative effort rate		
	Groups with 2+ restarts	Groups with 3+ restarts	Groups with 4+ restarts	Groups with 2+ restarts	Groups with 3+ restarts	Groups with 4+ restarts
Effect of 1st restart	1.721<br>(0.489)<br>[p=0.0004]	1.343<br>(0.540)<br>[p=0.0130]	1.360<br>(0.796)<br>[p=0.0874]	1.747<br>(0.675)<br>[p=0.0097]	1.684<br>(0.782)<br>[p=0.0313]	0.599<br>(0.911)<br>[p=0.5107]
Effect of 2nd restart	5.135<br>(0.915)<br>[p<0.0001]	3.022<br>(0.907)<br>[p=0.0009]	1.866<br>(1.213)<br>[p=0.1239]	0.119<br>(1.100)<br>[p=0.9141]	-0.049<br>(1.193)<br>[p=0.9672]	0.156<br>(1.617)<br>[p=0.9232]
Effect of 3rd restart		7.056<br>(1.230)<br>[p<0.0001]	2.323<br>(1.065)<br>[p=0.0292]		7.461<br>(1.397)<br>[p<0.0001]	5.318<br>(1.507)<br>[p=0.0004]
Effect of 4th restart			6.063<br>(1.727)<br>[p=0.0004]			9.467<br>(2.107)<br>[p<0.0001]
p-value 1st vs. 2nd	0.001	0.115	0.729	0.188	0.206	0.803
p-value 2nd vs. 3rd		0.009	0.777		0.000	0.019
p-value 3rd vs. 4th			0.046			0.063
Controls	N	N	N	N	N	N
N groups	741	462	197	741	462	197
N obs	9,636	9,858	5,998	9,636	9,858	5,998
R^2	0.0134	0.0181	0.0121	0.0010	0.0106	0.0167

Notes: Predicted restart effect estimated from OLS regressions of the group's round-to-round change in the financial contribution rate (Panel A) or cooperative effort rate (Panel B). The sample includes observations only from rounds that are part of a restart, and each column restricts the sample to groups that go through a given number of restarts. For example, the effect of the 1st restart is estimated by comparing the average round-to-round difference in cooperation rates for all rounds in cycle 1 to the difference in cooperation rates between the last round of cycle 1 and the first round of cycle 2, considering only groups that continue from cycle 1 to cycle 2. Similarly, column "Groups with 2+ restarts" includes only groups that participate in at least three cycles, and thus go through at least two restarts. Regressors are an indicator for the restart round, the restart number as a categorial variable, the interaction of the two, and groups fixed effects. Standard errors clustered at the group level in parentheses, and p-values from two-sided t-tests of the coefficient estimates in brackets.

Supplementary Table 4. Change in the size of the restart effect across cycles for fixed samples of groups, with controls

	A. Financial contribution rate			B. Cooperative effort rate		
	Groups with 2+ restarts	Groups with 3+ restarts	Groups with 4+ restarts	Groups with 2+ restarts	Groups with 3+ restarts	Groups with 4+ restarts
Effect of 1st restart	2.189<br>(0.794)<br>[p=0.0058]	3.244<br>(0.779)<br>[p<0.0001]	1.688<br>(0.867)<br>[p=0.0514]	4.139<br>(1.113)<br>[p=0.0002]	3.579<br>(0.914)<br>[p<0.0001]	0.559<br>(1.020)<br>[p=0.5835]
Effect of 2nd restart	4.839<br>(1.291)<br>[p=0.0002]	5.695<br>(1.250)<br>[p<0.0001]	2.097<br>(1.460)<br>[p=0.1508]	2.753<br>(1.406)<br>[p=0.0502]	2.052<br>(1.448)<br>[p=0.1565]	-0.128<br>(1.946)<br>[p=0.9477]
Effect of 3rd restart		8.756<br>(1.563)<br>[p<0.0001]	1.895<br>(1.104)<br>[p=0.0862]		8.692<br>(1.550)<br>[p<0.0001]	3.839<br>(1.465)<br>[p=0.0088]
Effect of 4th restart			6.157<br>(2.025)<br>[p=0.0024]			7.715<br>(2.283)<br>[p=0.0007]
p-value 1st vs. 2nd	0.022	0.031	0.786	0.320	0.280	0.718
p-value 2nd vs. 3rd		0.047	0.901		0.000	0.088
p-value 3rd vs. 4th			0.038			0.112
Controls	Y	Y	Y	Y	Y	Y
N groups	741	462	197	741	462	197
N obs	9,636	9,858	5,998	9,636	9,858	5,998
R^2	0.0252	0.0276	0.0163	0.0222	0.0248	0.0319

Notes: Predicted restart effect estimated from OLS regressions of the group's round-to-round change in the financial contribution rate (Panel A) or cooperative effort rate (Panel B). The sample includes observations only from rounds that are part of a restart, and each column restricts the sample to groups that go through a given number of restarts. For example, the effect of the 1st restart is estimated by comparing the average round-to-round difference in cooperation rates for all rounds in cycle 1 to the difference in cooperation rates between the last round of cycle 1 and the first round of cycle 2, considering only groups that continue from cycle 1 to cycle 2. Similarly, column “Groups with 2+ restarts” includes only groups that participate in at least three cycles, and thus go through at least two restarts. Regressors are an indicator for the restart round, the restart number as a categorial variable, the interaction of the two, and groups fixed effects. All regressions control for loan size, loan duration, calendar year fixed effects, and rainy season fixed effects. Standard errors clustered at the group level in parentheses, and p-values from two-sided t-tests of the coefficient estimates in brackets.

Supplementary Table 5. Cooperation rates at the start of a cycle in group lending

	Financial Contribution Rate		Cooperative Effort Rate	
	All Groups	5 Cycle Groups	All Groups	5 Cycle Groups
	(1)	(2)	(3)	(4)
Cycle	-0.375<br>(0.613)<br>[p=0.5410]	-0.357<br>(0.329)<br>[p=0.2795]	-2.423<br>(1.275)<br>[p=0.0576]	-5.326<br>(1.404)<br>[p=0.0002]
Controls	Y	Y	Y	Y
Group FE	Y	Y	Y	Y
N groups	1,589	197	1,589	197
N obs	4,015	985	4,015	985
R^2	0.3716	0.2186	0.5089	0.3257

Notes: Coefficient estimates from OLS regressions estimating the group's cooperation rate (financial contribution rate or cooperative effort rate) in the first round of a cycle. The sample is restricted to the first round of each cycle. The sample includes all groups in columns "All Groups" and groups that survive 5 cycles in columns "5 Cycle Groups". The regressor "Cycle" is the loan cycle number as a continuous variable. All regressions control for loan officer, loan size, loan duration, calendar year fixed effects, rainy season fixed effects, and group fixed effects. Standard errors clustered at the group level in parentheses, and p-values from two-sided t-tests of the coefficient estimates in brackets.

Supplementary Table 6. Interview sample client group-level descriptive statistics

Group ID	Group Size	Female[1]	Married[1]	Num Children[2]	Monthly Sales[3]	Loan Amount[3]	Loan Cycle[4]	Days Overdue[5]
G1	5	0.80	0.80	5.60	1.13	0.50	1	7
G2	5	1.00	1.00	2.00	0.88	0.80	1	0
G3	5	1.00	1.00	2.00	0.88	0.50	1	14
G4	5	1.00	1.00	3.00	0.86	0.50	1	7
G5	5	1.00	1.00	3.00	0.88	0.80	1	0
G6	5	1.00	1.00	2.40	0.89	0.50	2	13
G7	3	0.67	1.00	4.67	0.33	1.20	5	28
G8	5	1.00	1.00	2.20	1.05	0.80	1	0
G9	5	1.00	1.00	5.20	1.09	0.92	4	6
G10	4	1.00	1.00	4.00	1.18	0.80	2	30+
G11	4	1.00	1.00	5.25	0.36	1.00	5	0
G12	5	1.00	0.80	2.00	0.30	0.80	5	13
G13	5	0.80	1.00	3.40	0.93	1.00	5	7
G14	4	1.00	1.00	3.75	0.74	0.80	1	0
G15	5	1.00	1.00	2.60	0.88	0.80	1	0
G16	3	1.00	1.00	3.67	0.87	0.60	2	8
G17	5	0.80	1.00	2.00	0.87	0.80	2	8
G18	5	1.00	1.00	2.60	0.86	0.50	1	7
G19	4	1.00	1.00	2.25	0.91	0.50	1	30+
G20	3	1.00	1.00	2.33	0.80	0.50	2	3
G21	5	0.80	1.00	3.00	0.27	0.50	5	3
G22	5	0.80	0.80	2.00	0.86	0.50	1	3
G23	5	1.00	1.00	3.20	0.92	0.80	2	1
G24	5	1.00	1.00	3.60	1.00	0.30	1	30+
G25	5	1.00	1.00	2.40	1.21	1.30	5	0
G26	2	0.50	1.00	2.00	1.08	0.80	2	21
G27	4	1.00	1.00	2.25	0.43	0.80	4	30+
G28	3	1.00	0.66	3.33	0.46	0.80	4	0
G29	5	0.60	1.00	3.00	1.02	0.80	2	30+
G30	5	1.00	1.00	1.40	1.20	0.50	1	0
G31	4	1.00	0.75	2.50	0.78	1.00	3	23
G32	5	1.00	1.00	3.20	1.00	0.50	2	30+
G33	5	0.80	0.60	3.60	1.05	1.00	5	25
G34	5	0.60	1.00	3.00	0.66	0.80	4	0
G35	5	1.00	1.00	2.60	0.88	0.80	2	12

Notes: Group demographics are those reported prior to the first loan disbursement. [1]Proportion of the group members with the corresponding characteristic. [2]Average per group member. [3]Average per group member in millions of SLL. [4]Current or most recent loan cycle at the time of interview. [5]Final days overdue for the last completed loan cycle at the time of interview.

Supplementary Table 7. Interview sample staff descriptive statistics

Staff ID[1]	Staff Role[2]	Count	Years at MFI[3]	Age[4]	Gender[5]	Ethnic Group	Language[6]
S1 - S9 (Randomized)	Loan Officer	3	4.0	29	0.7	Kuranko, Temne, Temne	English
	Information & Accounting	2	7.0	36	0.0	Fullah, Mende	English
	Loan Portfolio Manager	2	4.5	37	0.0	Mende, Mende	English
	Executive Director	2	6.0	49	0.5	Krio, Undisclosed	English
Total		9	5.2	36	0.3		

Notes: Descriptive statistics of self-reported values at the time interviews were conducted, aggregated at the role level. [1]Staff IDs S1-S9 are randomized across roles to preserve confidentiality. [2]All staff interviewed, except one, had direct experience working as a loan officer for joint-liability groups. [3]Average years worked at the specific microfinance institution of this study. [4]Average age. [5]Proportion of women. [6]Interview language at the preference of the staff member.

Supplementary Table 8. Last-round effects on cooperation rates within loan cycles

	Financial Contribution Rate		Cooperative Effort Rate	
	Last Round	Penultimate Round	Last Round	Penultimate Round
Last round	-1.886<br>(0.384)<br>[p<0.0001]		1.859<br>(0.371)<br>[p<0.0001]	
Penultimate round		-3.085<br>(0.304)<br>[p<0.0001]		-2.783<br>(0.314)<br>[p<0.0001]
Intercept	100.568<br>(0.603)<br>[p<0.0001]	101.802<br>(0.552)<br>[p<0.0001]	93.145<br>(0.608)<br>[p<0.0001]	95.269<br>(0.571)<br>[p<0.0001]
Group FE	Y	Y	Y	Y
Cycle FE	Y	Y	Y	Y
N groups	1,587	1,587	1,587	1,587
N obs	8,026	8,026	8,026	8,026
R^2	0.1286	0.1308	0.2207	0.2522

Notes: Coefficient estimates from OLS regressions estimating the group's financial contribution rate and cooperative effort rate in the round. Columns "Last Round" regress the outcome on an indicator that the round is the last in a cycle with observations restricted to the last and penultimate rounds in a cycle. For comparison, columns "Penultimate Round" regress the outcome on an indicator that the round is the penultimate in a cycle with observations restricted to the penultimate and antepenultimate rounds in a cycle. In all columns, regressions include no controls other than loan cycle fixed effects and group fixed effects. Standard errors clustered at the group level in parentheses, and p-values from two-sided t-tests of the coefficient estimates in brackets.

Supplementary Table 9. Covariates of cooperation rates over time in group lending

	Financial Contribution Rate		Cooperative Effort Rate	
	Average Cycle 1	RE All Rounds	Average Cycle 1	RE All Rounds
Cycle duration[1]	0.828	-0.684	-1.995	0.130
	(0.704)	(0.506)	(1.342)	(0.564)
	[p=0.2394]	[p=0.1766]	[p=0.1373]	[p=0.8180]
Group size	-0.389	-1.467	-1.023	-1.690
	(0.326)	(0.341)	(0.541)	(0.466)
	[p=0.2325]	[p<0.0001]	[p=0.0588]	[p=0.0003]
Prop. female[2]	-0.686	-0.121	1.714	2.020
	(0.567)	(0.856)	(1.023)	(1.253)
	[p=0.2266]	[p=0.8873]	[p=0.0940]	[p=0.1069]
Prop. married[2]	-1.418	0.020	-3.745	-1.492
	(1.132)	(1.790)	(1.951)	(2.782)
	[p=0.2104]	[p=0.9909]	[p=0.0552]	[p=0.5918]
Avg. num. children[3]	0.314	0.545	0.812	1.160
	(0.200)	(0.210)	(0.320)	(0.365)
	[p=0.1167]	[p=0.0094]	[p=0.0112]	[p=0.0015]
Avg. monthly sales[4]	0.270	-3.218	1.484	-3.095
	(1.090)	(1.184)	(1.909)	(1.278)
	[p=0.8044]	[p=0.0066]	[p=0.4369]	[p=0.0154]
SD monthly sales[4]	-0.324	0.933	-1.759	1.199
	(0.824)	(0.971)	(1.511)	(1.006)
	[p=0.6947]	[p=0.3368]	[p=0.2445]	[p=0.2332]
Avg. business equity[4]	0.243	1.047	1.108	2.397
	(1.065)	(1.143)	(1.666)	(1.256)
	[p=0.8193]	[p=0.3597]	[p=0.5059]	[p=0.0562]
SD business equity[4]	-0.107	-0.990	-0.967	-1.849
	(0.641)	(0.904)	(1.063)	(0.996)
	[p=0.8674]	[p=0.2732]	[p=0.3630]	[p=0.0633]
Business diversity[5]	0.101	0.338	0.316	0.596
	(0.264)	(0.422)	(0.546)	(0.557)
	[p=0.7027]	[p=0.4234]	[p=0.5636]	[p=0.2846]
Loan size per member[4]	0.703	0.207	1.342	-2.074
	(0.553)	(0.531)	(1.115)	(0.694)
	[p=0.2039]	[p=0.6974]	[p=0.2289]	[p=0.0028]
Controls	Y	Y	Y	Y
N groups	1,589	1,589	1,589	1,589
N obs	1,589	31,199	1,589	31,199
R^2	0.1572	0.1348	0.2605	0.1963

Notes: Coefficient estimates. Columns "Average Cycle 1" are OLS regressions of the group's average

financial contribution rate and cooperative effort rate in cycle 1, with one observation per group. Columns "RE All Rounds" are random-effects regressions estimating the group's financial contribution rate or cooperative effort rate in the round, including all rounds. [1]In months. [2]Proportion of the group members with the corresponding characteristic. [3]Average per group member. [4]Average per group member in millions of SLL. [5]An index of business type diversity within the group normalized to mean of zero and standard deviation of one. Additional controls are branch, loan officer, calendar year fixed effects, and seasonal fixed effects. Round number, cycle number, and their interaction are included in RE All Rounds columns. For observations with missing covariate data, values were imputed using mean substitution. Standard errors clustered at the group level in parentheses, and p-values from two-sided t-tests of the coefficient estimates in brackets.

Supplementary Table 10. Cycle-to-cycle transition frequency

Group Size Cycle 1 (rows) → Group Size Cycle 2 (columns)

Group Size Cycle 1	-1	0	2	3	4	5	6	N
2	0.20	0.13	0.60	0.07	0.00	0.00	0.00	15
3	0.11	0.43	0.08	0.37	0.01	0.00	0.00	220
4	0.14	0.49	0.02	0.06	0.28	0.00	0.00	369
5	0.08	0.13	0.01	0.02	0.06	0.70	0.00	975
6	0.00	0.00	0.10	0.10	0.20	0.10	0.50	10

Group Size Cycle 2 (rows) → Group Size Cycle 3 (columns)

Group Size Cycle 2	-1	0	2	3	4	5	6	N
2	0.20	0.38	0.38	0.03	0.03	0.00	0.00	40
3	0.19	0.35	0.07	0.37	0.01	0.02	0.00	129
4	0.17	0.18	0.08	0.11	0.45	0.01	0.00	165
5	0.16	0.04	0.02	0.04	0.06	0.69	0.00	687
6	0.20	0.00	0.00	0.00	0.00	0.00	0.80	5

Group Size Cycle 3 (rows) → Group Size Cycle 4 (columns)

Group Size Cycle 3	-1	0	2	3	4	5	6	N
2	0.17	0.35	0.46	0.02	0.00	0.00	0.00	52
3	0.21	0.15	0.11	0.53	0.00	0.00	0.00	92
4	0.24	0.15	0.05	0.17	0.39	0.00	0.00	114
5	0.25	0.11	0.03	0.08	0.07	0.46	0.00	479
6	0.25	0.00	0.00	0.00	0.00	0.50	0.25	4

Group Size Cycle 4 (rows) → Group Size Cycle 5 (columns)

Group Size Cycle 4	-1	0	2	3	4	5	6	N
2	0.27	0.38	0.35	0.00	0.00	0.00	0.00	55
3	0.25	0.30	0.10	0.32	0.02	0.00	0.00	105
4	0.26	0.29	0.06	0.03	0.36	0.00	0.00	78
5	0.45	0.13	0.06	0.08	0.05	0.24	0.00	223
6	0.00	0.00	0.00	0.00	1.00	0.00	0.00	1

Notes: Values indicate the proportion of groups that become of a given group size in the subsequent cycle (columns) conditional on the group size in the current cycle (rows). Column N indicates the conditional sample size. Transitioning to "-1" means that the group is not observed in the subsequent cycle and the group did not repay in full all rounds in the current cycle. Transitioning to "0" means that the group is not observed in the subsequent cycle and the group repaid in full all rounds in the current cycle. Red shade indicates transitions to "-1," blue shade indicates transitions to "0," yellow shade indicates decreases in group size, no shade indicates no changes in group size, and green shade indicates increases in group size.

Supplementary File

Supplementary File 1. Protocol for semi-structured client interviews
Prompts and example questions by topic.

1. **Introductions and Informed Consent:**
   - Local greeting and introductions. [Researcher, client, and translator.]
   - Selection of preferred language for the client.
   - Researcher background and context.
   - Research purpose, data use, interview length, and informed consent.
   - If you do not want to answer a question, you do not have to answer.
   - Do you have any questions for me before we start? [Respond to any interviewee questions.]

[Begin audio recording.]

2. **Background:** [Warmup.]
   - What is your name?
   - What [ethnic group] do you belong to?
   - What do you do for work?
   - Can you describe where you spend your time during a normal day?

3. **Group Lending Verification:** [Confirm if basic lending information and data are correct.]
   - What is the name of your lending group?
   - Do you currently have a loan?
   - How many loans have you taken from [the lending institution]?
   - How many members are in your group?
   - What are the names of your group members?
   - Confirm basic repayment information [e.g. the group has paid back two loans in full and just received the disbursement for the third loan last month.]

4. **Social Connections Within Group:** [Understand how the group members know each other.]
   - How do you know each of the group members?
   - How long have you known each other?
   - Did you know each other before joining the credit group?
   - What was most important when you decided who you wanted in your group?
   - How often do you see each other?
   - What activities do you do together?

5. **Client Perception of the Dilemma Structure and Responsibilities:** [Probe client understanding of the joint liability dilemma and consequences.]
   - How much did you and your group members receive in the loan?
   - What happens if you do not repay the loan?
   - What happens if one of your group members do not repay the loan?

6. **Repayment Process:** [Understand the practical logistics of repayment.]
   - How do you collect the money and make the repayment?
   - After you received the loan, how often and why would you see each member of the group?
   - Would you meet as a group? How often?
   - How do you get the money to pay the loan?
   - What do you do if you do not have the money on time?

7. **Cooperative Motivation and Incentives:** [Collect self-report reasons for repayment.]
   - Why do you choose to repay the loan?
   - Did you ever pay for another member? If yes, when?
   - Why did you pay for the other member?
   - Why do the members of your group choose to repay the loan?

8. **Free-riding and/or Cooperative Behavior:** [Explore extent and reasons for paying or not paying.]
   - Did you ever not make a payment? If so, when and why?
   - If you were having difficulty repaying, what did you do?
   - Did any of the other members pay for you? If so, when and why?
   - Did you repay them? If yes, when and how?
   - Did any other members of the group not make a payment? If so, when and why?
   - Did you ever pay for another member? If so, when and why?
   - Did you expect them to repay you? Did they repay you? How?
   - How do you know if a member has money to pay or not?
   - How many times are you willing to pay for another member?

9. **Loan Enforcement:** [Explore informal and formal enforcement mechanisms.]
   - How do you know if a member is not going to pay?
   - What would you do if a member is not going to pay?
   - How did the group respond if someone is not going to pay?
   - Did the person who did not want to pay eventually pay?
   - When you were not able to repay, what did the other members of the group do?
   - What does the lending institution do if someone does not pay?

10. **Behavioral, Financial, and Structural Changes Over Time:** [Explore what underlies the temporal patterns.]
   - Refer the interviewee to their specific group repayment history.
   - Why did the group pay late here? Why were things difficult here?
   - Why did the group pay early or on-time here? Why did things go well here?
   - If different repayment patterns for the group, what changed?
   - If a specific member did not want to repay, why do you think the group member did not want to pay anymore?
   - If you had a previous loan, how was your previous loan different from this loan?
   - Did you find your first or last loan easier to repay? Why?
   - Has there been any change in group membership over time? If so, when and why?
   - What happened at the end of this loan for the group? Who paid and why?

- What happened to any of the members that did not repay?
- How often do you now see the members that did not repay?
- Did a member not repaying change your relationship with them? If so, how?

11. Closing: [Allocate time for ending the interviewing and responding to questions.]

- Thank the interviewee for their time.
- Do you have any more questions for me? [Respond to questions.]

[End audio recording.]

Supplementary References

1. Dawes, R. M. Social Dilemmas. *Annu. Rev. Psychol.* **31**, 169–193 (1980).
2. Ledyard, J. Public Goods: A Survey of Experimental Research. in *The Handbook of Experimental Economics* (eds. Kagel, J. & Roth, A.) (Princeton University Press, Princeton, 1995).
3. Diekmann, A. Volunteer's dilemma. *Journal of conflict resolution* **29**, 605–610 (1985).
4. Hovi, J. Binary games as models of public goods provision. *Scan. Polit. Stud.* **9**, 337–360 (1986).
5. Kollock, P. Social dilemmas: The Anatomy of Cooperation. *Annu. Rev. Sociol.* **24**, 182–214 (1998).
6. Skyrms, B. *The Stag Hunt and the Evolution of Social Structure*. (Cambridge University Press, 2004).
7. McAdams, R. H. Beyond the prisoners' dilemma: Coordination, game theory, and law. *S. Cal. L. Rev.* **82**, 209 (2008).
8. Archetti, M. & Scheuring, I. Coexistence of cooperation and defection in public goods games. *Evolution (N Y).* **65**, 1140–1148 (2011).
9. Archetti, M. & Scheuring, I. Game theory of public goods in one-shot social dilemmas without assortment. *J. Theor. Biol.* **299**, 9–20 (2012).
10. Taylor, M. & Ward, H. Chickens, whales, and lumpy goods: alternative models of public-goods provision. *Polit. Stud. (Oxf).* **30**, 350–370 (1982).
11. Hardin, R. *Collective Action*. (Resources for the Future Press, 1982).
12. Raihani, N. J. & Bshary, R. The evolution of punishment in n-player public goods games: A volunteer's dilemma. *Evolution (N Y).* **65**, 2725–2728 (2011).
13. Przepiorka, W. & Diekmann, A. Individual heterogeneity and costly punishment: a volunteer's dilemma. *Proceedings of the Royal Society B: Biological Sciences* **280**, (2013).
14. Bach, L. A., Helvik, T. & Christiansen, F. B. The evolution of n-player cooperation—threshold games and ESS bifurcations. *J. Theor. Biol.* **238**, 426–434 (2006).
15. Archetti, M. The volunteer's dilemma and the optimal size of a social group. *J. Theor. Biol.* **261**, 475–480 (2009).
16. Van de Kragt, A. J. C., Orbell, J. M. & Dawes, R. M. The minimal contributing set as a solution to public goods problems. *American Political Science Review* **77**, 112–122 (1983).
17. Palfrey, T. R. & Rosenthal, H. Participation and the provision of discrete public goods: a strategic analysis. *J. Public Econ.* **24**, 171–193 (1984).
18. Bagnoli, M. & Lipman, B. L. Provision of Public Goods: Fully Implementing the Core through Private Contributions. *Rev. Econ. Stud.* **56**, 583–601 (1989).
19. Isaac, R. M., Schmidtz, D. & Walker, J. M. The assurance problem in a laboratory market. *Public Choice* **62**, 217–236 (1989).
20. Bagnoli, M. & McKee, M. Voluntary contribution games: Efficient private provision of public goods. *Econ. Inq.* **29**, 351–366 (1991).
21. Suleiman, R. & Rapoport, A. Provision of step-level public goods with continuous contribution. *J. Behav. Decis. Mak.* **5**, 133–153 (1992).
22. Croson, R. & Marks, M. Identifiability of individual contributions in a threshold public goods experiment. *J. Math. Psychol.* **42**, 167–190 (1998).

23. Marks, M. & Croson, R. Alternative rebate rules in the provision of a threshold public good: An experimental investigation. *J. Public Econ.* **67**, 195–220 (1998).
24. Cadsby, C. B. & Maynes, E. Voluntary provision of threshold public goods with continuous contributions: experimental evidence. *J. Public Econ.* **71**, 53–73 (1999).
25. Coats, J. C., Gronberg, T. J. & Grosskopf, B. Simultaneous versus sequential public good provision and the role of refunds—an experimental study. *J. Public Econ.* **93**, 326–335 (2009).
26. Schelling, T. C. *The Strategy of Conflict*. (Harvard university press, 1980).
27. Diekmann, A. & Przepiorka, W. "Take one for the team!" individual heterogeneity and the emergence of latent norms in a volunteer's dilemma. *Social Forces* **94**, 1309–1333 (2016).
28. Dawes, R. M., Orbell, J. M., Simmons, R. T. & Van De Kragt, A. J. C. Organizing groups for collective action. *American Political Science Review* **80**, 1171–1185 (1986).
29. Normann, H.-T. & Rau, H. A. Simultaneous and sequential contributions to step-level public goods: One versus two provision levels. *Journal of Conflict Resolution* **59**, 1273–1300 (2015).
30. Olson, M. *The Logic of Collective Action: Public Goods and the Theory of Groups*. (Harvard University Press, Cambridge, 1965).
31. Fehr, E. & Schurtenberger, I. Normative foundations of human cooperation. *Nat. Hum. Behav.* **2**, 458–468 (2018).
32. Bruhin, A. *et al.* Understanding Mechanisms of Persistence in Prosocial Behavior: Evidence from a Large-Scale Field Experiment. *Journal of Political Economy Microeconomics* (2025).
33. Allcott, H. & Rogers, T. The Short-Run and Long-Run Effects of Behavioral Interventions: Experimental Evidence from Energy Conservation. *American Economic Review* **104**, 3003–3037 (2014).
34. Sabin, N. & Reed-Tsochas, F. Able but Unwilling to Enforce: Cooperative Dilemmas in Group Lending. *American Journal of Sociology* **125**, 1602–1667 (2020).
35. Oliver, P. Rewards and punishments as selective incentives for collective action: theoretical investigations. *American journal of sociology* **85**, 1356–1375 (1980).
