# Supplementary material for: Punctuated decline of human cooperation
Source: Nature. 2026 Apr 22;653(8116):1110–8. doi: 10.1038/s41586-026-10380-3 (PMC13215883; doi:10.1038/s41586-026-10380-3)
Supplement: Supplementary file 2 — Reporting Summary [file 41586_2026_10380_MOESM2_ESM.pdf]

nature portfolio

Corresponding author(s): Nicholas Sabin

Last updated by author(s): Mar 7, 2026

Reporting Summary

Nature Portfolio wishes to improve the reproducibility of the work that we publish. This form provides structure for consistency and transparency in reporting. For further information on Nature Portfolio policies, see our Editorial Policies and the Editorial Policy Checklist.

Statistics

For all statistical analyses, confirm that the following items are present in the figure legend, table legend, main text, or Methods section.

n/a	Confirmed	
☐	☒	The exact sample size (*n*) for each experimental group/condition, given as a discrete number and unit of measurement
☐	☒	A statement on whether measurements were taken from distinct samples or whether the same sample was measured repeatedly
☐	☒	The statistical test(s) used AND whether they are one- or two-sided<br>*Only common tests should be described solely by name; describe more complex techniques in the Methods section.*
☐	☒	A description of all covariates tested
☐	☒	A description of any assumptions or corrections, such as tests of normality and adjustment for multiple comparisons
☐	☒	A full description of the statistical parameters including central tendency (e.g. means) or other basic estimates (e.g. regression coefficient) AND variation (e.g. standard deviation) or associated estimates of uncertainty (e.g. confidence intervals)
☐	☒	For null hypothesis testing, the test statistic (e.g. *F*, *t*, *r*) with confidence intervals, effect sizes, degrees of freedom and *P* value noted<br>*Give P values as exact values whenever suitable.*
☒	☐	For Bayesian analysis, information on the choice of priors and Markov chain Monte Carlo settings
☐	☒	For hierarchical and complex designs, identification of the appropriate level for tests and full reporting of outcomes
☐	☒	Estimates of effect sizes (e.g. Cohen's *d*, Pearson's *r*), indicating how they were calculated

Our web collection on statistics for biologists contains articles on many of the points above.

Software and code

Policy information about availability of computer code

Data collection	The quantitative data was originally collected for the purpose of administering microfinance services in Sierra Leone from 2005 to 2011. The data were electronically recorded by the lending and accounting staff in the organization's Management Information System (MIS). The quantitative data were recorded independently of the research team.
Data analysis	For the quantitative data, the statistical analysis and figure generation were conducted using STATA 18.5, including package Markstat 2.1, and the software R version 4.2.3.<br><br>For the interview data, audio recordings of the interviews were manually transcribed verbatim by the research team. The data was managed and coded using NVivo software version 14.24.1.<br><br>Code to reproduce the statistical analysis and figure source data are publicly available at the Open Science Framework (https://doi.org/10.17605/OSF.IO/26BFC)

For manuscripts utilizing custom algorithms or software that are central to the research but not yet described in published literature, software must be made available to editors and reviewers. We strongly encourage code deposition in a community repository (e.g. GitHub). See the Nature Portfolio guidelines for submitting code & software for further information.

Data

Policy information about availability of data

All manuscripts must include a data availability statement. This statement should provide the following information, where applicable:

- Accession codes, unique identifiers, or web links for publicly available datasets
- A description of any restrictions on data availability
- For clinical datasets or third party data, please ensure that the statement adheres to our policy

The deidentified group lending data is publicly available at the Open Science Framework (https://doi.org/10.17605/OSF.IO/26BFC)

Research involving human participants, their data, or biological material

Policy information about studies with human participants or human data. See also policy information about sex, gender (identity/presentation), and sexual orientation and race, ethnicity and racism.

Reporting on sex and gender	The data includes information on self-reported gender as recorded by the lending institution during the loan application process. Consistent with the organization's social objectives, 73.3% of the clients are self-reported female. Regression analysis in the supplementary information includes the proportion of female clients in a group as a covariate. In the main text, the econometric model employs group fixed effects such that time-consistent group-level covariates are not necessary.
Reporting on race, ethnicity, or other socially relevant groupings	Self-reported ethnic group was recorded in the interview sample. Anonymized descriptive statistics for the interview sample are included in the Extended Data.<br><br>We do not include data on race, ethnicity, or other socially relevant groupings in the quantitative analysis. In the supplemental analyses, we do include direct economic measures of clients' monthly sales and business equity as reported to and verified by the lending institution.
Population characteristics	See below in "Research sample."
Recruitment	The quantitative data sample is limited to clients in Sierra Leone that received a joint-liability loan at the microfinance institution described in the study. Consistent with the organization's focus on poverty alleviation, all clients are low-income. Furthermore, microcredit group members must meet basic eligibility criteria. Specifically, each client is required to have their own micro-business capable of meeting the minimum loan repayments and members of the same group cannot be direct kin, i.e., parents, spouses, or siblings. The sample is representative of the population of joint-liability clients at the microfinance institution. However, please note that microfinance clients are not representative of the adult population of Sierra Leone more broadly, given that individuals self-select into applying for loans at the microfinance institution. Therefore, results do not necessarily generalize to the adult population of Sierra Leone more broadly, and we make no claims in the paper of such generalizability.<br><br>The sample of clients for interviews was drawn from the overall quantitative dataset, using a two-stage cluster random sampling, plus a purposive enhancement. The sample of clients for interviews is representative of the quantitative data sample, but, as stated previously, cannot be seen as being representative of the adult population of Sierra Leone more broadly, and we make no claims in the paper of such generalizability. In the first stage of the random sampling, we used simple randomization of groups, after restricting the population of potential groups based on two criteria: (i) we geographically restricted the pool to groups that were administered at the lending institution's principal branch. This was implemented for practical efficiency of interview logistics; (ii) we restricted the pool to groups that had been engaged in borrowing within the last six months. This was implemented to reduce recall bias during the interviews. This resulted in 35 joint liability groups drawn by simple randomization from the subpopulation. Supplementary Table 6 provides descriptive statistics of the interview sample at the group-level. In the second stage of the random sampling, we selected one member per group to be interviewed using simple randomization within the group. We then enhanced this sampling design by implementing a purposive sampling of an additional member from within the randomly selected groups. The choice of whether to conduct an additional interview and with which specific member was based on the content provided in the first member's interview, following the researcher's discretion regarding which additional group member's perspective would provide the most valuable information. For example, if the first interviewee indicated that a specific member "X" had been the main source of cooperative disruption in the group, member X was selected for a direct interview to hear his or her version of the events. The intent of additional within-group interviews was to cross-validate the initial interview, collect potentially contradictory data, and understand a complex phenomenon from different points of view. This resulted in 29 additional interviews, producing a total of 64 client interviews. Extended Data Table 3 provides descriptive statistics of the client interview sample at the individual-level. Interviews were also conducted with a non-random sample of 9 staff members of the lending institution – including three loan officers, two information and accounting officers, two loan portfolio managers, and two executive directors – regarding organization policies, practices in the field, and the organization's record keeping process. Supplementary Table 7 provides descriptive statistics of the staff interview sample. The interview content was instrumental in accurately interpreting the quantitative dataset and provided context and further cross-validation of the borrowers' descriptions.
Ethics oversight	This study conducts analysis of secondary data originally collected by the microfinance institution and primary interview data collected by the principal investigator. This research was approved by the Central University Research Ethics Committee

(CUREC) at the University of Oxford. Reference Number: SSD/CUREC1A/10-099. The approval included the collection process and analysis of administrative microfinance data and of primary data from human participants collected through semi-structured interviews. The research was performed in accordance with all relevant guidelines and regulations outlined by CUREC. The analysis and publication of the secondary administrative data follow the Data Use Agreement indicating that the research team may disclose and/or publish data such that "the data shall be anonymized and/or aggregated so that no reference is made to individuals' names, applying to both [microfinance institution] clients and staff." Before each interview during primary data collection, the research purpose and use of the interview data was explained to the client or staff member and a physical document of informed consent was reviewed together. All participants provided informed consent by signature or thumbprint. To safeguard participant confidentiality, personally identifiable information was redacted from the interview data.

Note that full information on the approval of the study protocol must also be provided in the manuscript.

Field-specific reporting

Please select the one below that is the best fit for your research. If you are not sure, read the appropriate sections before making your selection.

☐ Life sciences ☒ Behavioural & social sciences ☐ Ecological, evolutionary & environmental sciences

For a reference copy of the document with all sections, see nature.com/documents/nr-reporting-summary-flat.pdf

Behavioural & social sciences study design

All studies must disclose on these points even when the disclosure is negative.

Study description

A field study of long-term, high-stakes cooperative behavior that is based on the analysis of a longitudinal quantitative dataset and an interview-based qualitative dataset.

Research sample

The sample involves microfinance clients in Sierra Leone participating in group loans. The microfinance institution had approximately 18,000 borrowers at the time of data collection spread throughout multiple geographic regions in Sierra Leone spanning both urban and rural areas. The lending groups are small, typically five members, and are formed through a self-selection process. Loan officers ensure that each member meets basic eligibility criteria; most notably, each client is required to have their own micro-business capable of meeting the minimum loan repayments. Typical micro-business examples include petty trading, food service, barbershop, tailoring, motorbike taxi service. Members of the same group cannot be direct kin, i.e., parents, spouses, or siblings.

The quantitative data sample is representative of the population of joint-liability clients at the microfinance institution. However, please note that microfinance clients are not representative of the adult population of Sierra Leone more broadly, given that individuals select into applying for loans at the microfinance institution. Therefore, results do not necessarily generalize to the adult population of Sierra Leone more broadly, and we make no claims in the paper of such generalizability.

The rational for analyzing the quantitative research sample is that it provides a real-world social dilemma in which cooperative behavior with high stakes to the participants are tracked in the long-term. The social dilemma occurs because borrowers enter a joint-liability contract such that if the group loan is not repaid in full, all group members are held financially responsible regardless of who defaulted. The same dilemma is faced by group members on a monthly basis for up to five years. The quantitative data sample was chosen because it comprised all clients who received a joint-liability loan at the microfinance institution during the study period. 73.3% of the sample were female. 93.6% of the sample were married. The average number of children was 3.1. We have no information regarding age of the clients.

The rational for the qualitative research sample was to perform two key functions in the study: (1) the data help contextualize the cooperative dilemma, to ensure proper understanding of the quantitative patterns and their appropriate interpretation; (2) the data provide insight to the behavioral mechanisms underlying the longitudinal trends.

Sampling strategy

For the quantitative dataset, the sample includes all group loans administered by the microfinance institution during a period between 2005 and 2011, with minor data exclusions noted below. The dataset includes 47,931 group payments, partial or full, (corresponding to 31,199 scheduled monthly payments) made by 7,108 borrowers (constituting 1,589 unique lending groups) over five years. Sample size was not pre-determined. Models in the study document the statistical significance and confidence intervals of the empirical relationships. The cooperative dynamics in the main analysis are strongly significant with P-values less than 0.001.

For the interview dataset, the sample consists of 73 in-depth semi-structured interviews: 64 interviews with group lending clients and 9 interviews with members of the lending institution staff. The sample of clients for interviews was selected to be representative of the quantitative data sample. Moreover, it included a purposive second stage to capture perspectives by multiple clients involved in a given cooperative incident.

In the first stage of the random sampling, we used simple randomization of groups, after restricting the population of potential groups based on two criteria: (i) we geographically restricted the pool to groups that were administered at the lending institution's principal branch. This was implemented for practical efficiency of interview logistics; (ii) we restricted the pool to groups that had been engaged in borrowing within the last six months. This was implemented to reduce recall bias during the interviews. This resulted in 35 lending groups drawn by simple randomization from the subpopulation. In the second stage of the random sampling, we selected one member per group to be interviewed using simple randomization within the group. We then enhanced this sampling design by implementing a purposive sampling of an additional member from within the randomly selected groups. The choice of

whether to conduct an additional interview and with which specific member was based on the content provided in the first member's interview, following the researcher's discretion regarding which additional group member's perspective would provide the most valuable information. For example, if the first interviewee indicated that a specific member "X" had been the main source of cooperative disruption in the group, member X was selected for a direct interview to hear his or her version of the events. The intent of additional within-group interviews was to cross-validate the initial interview, collect potentially contradictory data, and understand a complex phenomenon from different points of view. This resulted in 29 additional interviews, producing a total of 64 client interviews. Interviews were also conducted with a non-random sample of 9 staff members of the lending institution, including three loan officers, two information and accounting officers, two loan portfolio managers, and two executive directors, regarding organization policies, practices in the field, and the organization's record keeping process.

Data collection	The quantitative data was collected by the lending institution for the primary purpose of administering microfinance services. Client demographics and group loan repayment behavior were electronically recorded in the organization's Management Information System (MIS) by staff. The research team was not involved with the quantitative data collection. The group repayments occur at the local branch office of the microfinance institution. It is not required that all group members be present when making a group payment.<br><br>The interview data is based on 73 in-depth semi-structured interviews. On average, client interviews lasted 39 minutes and staff interviews lasted 1 hour and 34 minutes. Interview time totaled 56 hours. The interviews were conducted in person in Sierra Leone by the principal investigator between April 5, 2011 and June 6, 2011, contemporaneous to the collection of the quantitative data. The principal investigator was not blinded to the purpose of the interview, which was to collect clients' perspectives and experiences.
Timing	The group loans were administered between 2005 and 2011 by the microfinance institution.
Data exclusions	The dataset includes all group loans of up to five loan cycles disbursed and scheduled to complete repayment during a period between 2005 and 2011. Loan cycles greater than five are not included in the analysis. The sample size decreases with each loan cycle, with few groups having taken more than five cycles (approximately 3% of groups). We restrict the analysis to the first five loan cycles because the sample size of the remaining cycles is not sufficient for statistical analysis of longitudinal trends. We exclude from the analysis three groups due to missing data on an antecedent cycle (e.g., existing data on loan cycle 1 and 3 but missing data on cycle 2).
Non-participation	After the clients of a group receive a loan disbursement, the dataset has a full record of each group's longitudinal repayment behavior. In addition to timely repayment, this record may include a group's late, partial, or complete lack of repayment (which may be conceptualized as "non-participation.") This information is used to measure each group's cooperation over time.
Randomization	The collection and construction of the quantitative data did not involve randomization, as we used the full sample of group-lending clients of the microfinance institution. Randomization was involved in two-stage clustered random sampling used to determine the set of clients to be interviewed. Randomization was implemented in spreadsheet software.

Reporting for specific materials, systems and methods

We require information from authors about some types of materials, experimental systems and methods used in many studies. Here, indicate whether each material, system or method listed is relevant to your study. If you are not sure if a list item applies to your research, read the appropriate section before selecting a response.

Materials & experimental systems

n/a	Involved in the study	
☒	☐	Antibodies
☒	☐	Eukaryotic cell lines
☒	☐	Palaeontology and archaeology
☒	☐	Animals and other organisms
☒	☐	Clinical data
☒	☐	Dual use research of concern
☒	☐	Plants

Methods

n/a	Involved in the study	
☒	☐	ChIP-seq
☒	☐	Flow cytometry
☒	☐	MRI-based neuroimaging

Plants

Seed stocks	*Report on the source of all seed stocks or other plant material used. If applicable, state the seed stock centre and catalogue number. If plant specimens were collected from the field, describe the collection location, date and sampling procedures.*
Novel plant genotypes	*Describe the methods by which all novel plant genotypes were produced. This includes those generated by transgenic approaches, gene editing, chemical/radiation-based mutagenesis and hybridization. For transgenic lines, describe the transformation method, the number of independent lines analyzed and the generation upon which experiments were performed. For gene-edited lines, describe the editor used, the endogenous sequence targeted for editing, the targeting guide RNA sequence (if applicable) and how the editor was applied.*
Authentication	*Describe any authentication procedures for each seed stock used or novel genotype generated. Describe any experiments used to assess the effect of a mutation and, where applicable, how potential secondary effects (e.g. second site T-DNA insertions, mosiacism, off-target gene editing) were examined.*
